# Supplementary material for: Controlling oncogenic KRAS signaling pathways with a Palladium-responsive peptide
Source: Commun Chem. 2022 Jun 23;5:75. doi: 10.1038/s42004-022-00691-7 (PMC9814687; doi:10.1038/s42004-022-00691-7)
Supplement: Supplementary file 2 — Reporting Summary [file 42004_2022_691_MOESM2_ESM.pdf]

nature research

Corresponding author(s): M. Eugenio Vázquez

Last updated by author(s): May 17, 2022

Reporting Summary

Nature Research wishes to improve the reproducibility of the work that we publish. This form provides structure for consistency and transparency in reporting. For further information on Nature Research policies, see our Editorial Policies and the Editorial Policy Checklist.

Statistics

For all statistical analyses, confirm that the following items are present in the figure legend, table legend, main text, or Methods section.

n/a	Confirmed	
☐	☒	The exact sample size (*n*) for each experimental group/condition, given as a discrete number and unit of measurement
☐	☒	A statement on whether measurements were taken from distinct samples or whether the same sample was measured repeatedly
☐	☒	The statistical test(s) used AND whether they are one- or two-sided<br>*Only common tests should be described solely by name; describe more complex techniques in the Methods section.*
☒	☐	A description of all covariates tested
☒	☐	A description of any assumptions or corrections, such as tests of normality and adjustment for multiple comparisons
☐	☒	A full description of the statistical parameters including central tendency (e.g. means) or other basic estimates (e.g. regression coefficient) AND variation (e.g. standard deviation) or associated estimates of uncertainty (e.g. confidence intervals)
☒	☐	For null hypothesis testing, the test statistic (e.g. *F*, *t*, *r*) with confidence intervals, effect sizes, degrees of freedom and *P* value noted<br>*Give P values as exact values whenever suitable.*
☒	☐	For Bayesian analysis, information on the choice of priors and Markov chain Monte Carlo settings
☒	☐	For hierarchical and complex designs, identification of the appropriate level for tests and full reporting of outcomes
☒	☐	Estimates of effect sizes (e.g. Cohen's *d*, Pearson's *r*), indicating how they were calculated

Our web collection on statistics for biologists contains articles on many of the points above.

Software and code

Policy information about availability of computer code

Data collection	All measures were collected using the manufacture's software for each of the instruments (e.g., fluorescence measurements with DataMax 2.20 supplied with the Jobin-Yvon Fluoromax-3 spectrophotometer).
Data analysis	Data analysis was performed with Prism 8 for Mac OS X (v. 8.4.3). Titration curve fitting was made using the DYNAFIT software package by BIOKIN (http://www.biokin.com/dynafit/). NMR data was processed with TopSpin 3.5 (Bruker 2020)

For manuscripts utilizing custom algorithms or software that are central to the research but not yet described in published literature, software must be made available to editors and reviewers. We strongly encourage code deposition in a community repository (e.g. GitHub). See the Nature Research guidelines for submitting code & software for further information.

Data

Policy information about availability of data

All manuscripts must include a data availability statement. This statement should provide the following information, where applicable:

- Accession codes, unique identifiers, or web links for publicly available datasets
- A list of figures that have associated raw data
- A description of any restrictions on data availability

The datasets generated and analyzed during the current study are available from the corresponding authors on reasonable request.

April 2020

Field-specific reporting

Please select the one below that is the best fit for your research. If you are not sure, read the appropriate sections before making your selection.

☒ Life sciences ☐ Behavioural & social sciences ☐ Ecological, evolutionary & environmental sciences

For a reference copy of the document with all sections, see nature.com/documents/nr-reporting-summary-flat.pdf

Life sciences study design

All studies must disclose on these points even when the disclosure is negative.

Sample size	*Describe how sample size was determined, detailing any statistical methods used to predetermine sample size OR if no sample-size calculation was performed, describe how sample sizes were chosen and provide a rationale for why these sample sizes are sufficient.*
Data exclusions	No data were excluded from our studies.
Replication	technical triplicates were performed.
Randomization	NOT relevant to our study. Appropriate controls were included in our experiments where necessary.
Blinding	NOT relevant to our study. This is a classic work in which we evaluate the effect of a synthesized probe. Blinding is not customary in such studies.

Reporting for specific materials, systems and methods

We require information from authors about some types of materials, experimental systems and methods used in many studies. Here, indicate whether each material, system or method listed is relevant to your study. If you are not sure if a list item applies to your research, read the appropriate section before selecting a response.

Materials & experimental systems

n/a	Involved in the study	
☐	☒	Antibodies
☐	☒	Eukaryotic cell lines
☒	☐	Palaeontology and archaeology
☒	☐	Animals and other organisms
☒	☐	Human research participants
☒	☐	Clinical data
☒	☐	Dual use research of concern

Methods

n/a	Involved in the study	
☒	☐	ChIP-seq
☒	☐	Flow cytometry
☒	☐	MRI-based neuroimaging

Antibodies

Antibodies used	Anti-ERK 1/2, Anti-p-ERK 1/2
Validation	Antibodies were purchased from Sta. Cruz Biotechnologies with catalog numbers:<br>ERK 1/2 Antibody (C-9): sc-514302<br>p-ERK 1/2 Antibody (pT202/pY204.22A): sc-136521

Eukaryotic cell lines

Policy information about cell lines

Cell line source(s)	A549
Authentication	None of the cell lines used were authenticated
Mycoplasma contamination	All cell lines tested negative in periodical routine tests performed in our lab
Commonly misidentified lines (See ICLAC register)	Commonly misidentified lines were NOT used in this study
