# Supplementary figures and images for: Controlling oncogenic KRAS signaling pathways with a Palladium-responsive peptide
Source: Commun Chem. 2022 Jun 23;5:75. doi: 10.1038/s42004-022-00691-7 (PMC9814687; doi:10.1038/s42004-022-00691-7)

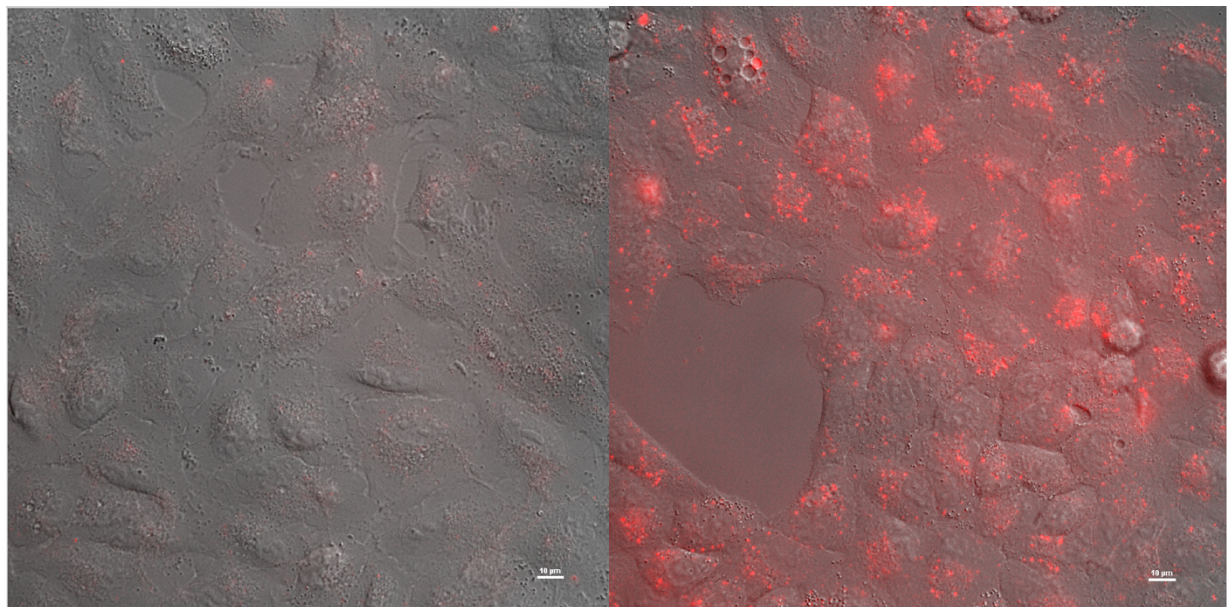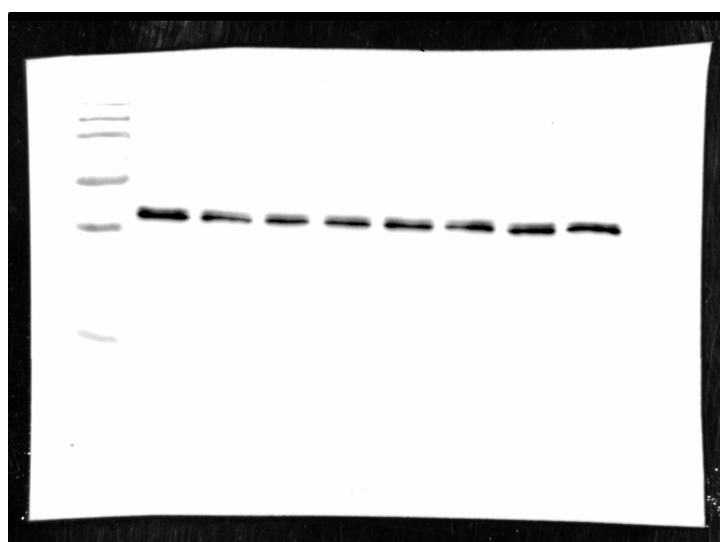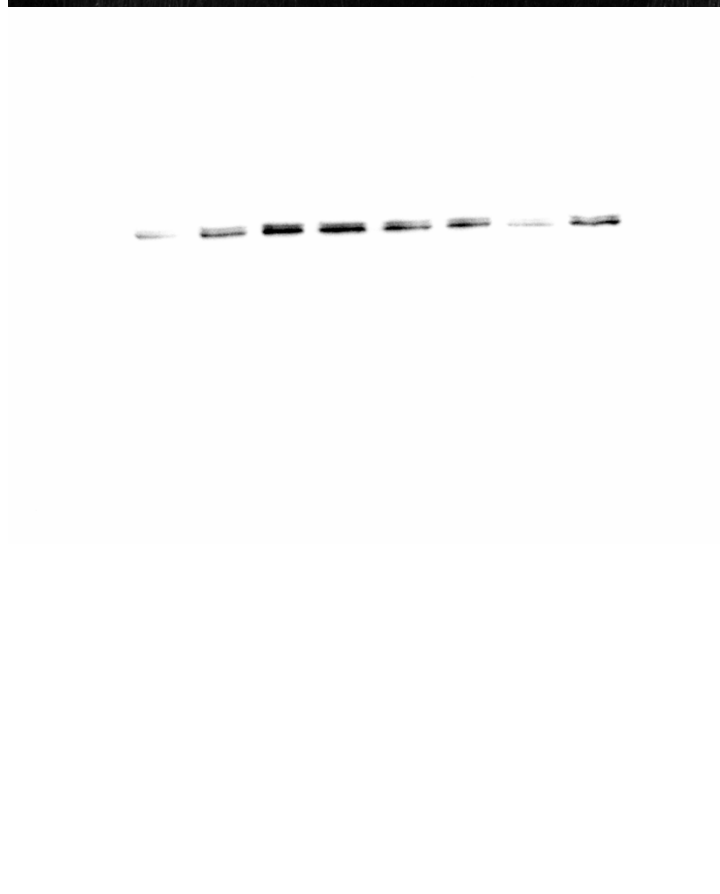

Supplement: Supplementary file 3 — Supplementary Data 2 [file 42004_2022_691_MOESM3_ESM.pdf]
