# Supplementary material for: Controlling oncogenic KRAS signaling pathways with a Palladium-responsive peptide
Source: Commun Chem. 2022 Jun 23;5:75. doi: 10.1038/s42004-022-00691-7 (PMC9814687; doi:10.1038/s42004-022-00691-7)
Supplement: Supplementary file 5 — Supplementary Information [file 42004_2022_691_MOESM5_ESM.pdf]

Controlling Oncogenic KRAS Signaling Pathways with a Palladium-Responsive Peptide

Soraya Learte-Aymamí,[1] Pau Martin-Malpartida,[2] Lorena Roldán-Martín,[3] Giuseppe Sciortino,[3,4] José R. Couceiro,[1] Jean-Didier Maréchal,[3] Maria J. Macias,[2,5] José L. Mascareñas,[1,*] M. Eugenio Vázquez,[1,*]

1 Centro Singular de Investigación en Química Biolóxica e Materiais Moleculares (CiQUS), Departamento de Química Orgánica, Universidade de Santiago de Compostela. Santiago de Compostela 15705, Spain.

2 Institute for Research in Biomedicine (IRB Barcelona), The Barcelona Institute of Science and Technology, Barcelona 08028, Spain.

3 Insilichem, Departament de Química, Universitat Autònoma de Barcelona, Cerdanyola 08193, Spain.

4 Institute of Chemical Research of Catalonia (ICIQ). The Barcelona Institute of Science and Technology, Tarragona 43007, Spain.

5 Institució Catalana de Recerca i Estudis Avançats (ICREA), Passeig Lluís Companys 23, Barcelona 08010, Spain.

.

Supplementary Note 1

CRediT Author attributions

The following table describes the contribution of each author according to the CRediT taxonomy as described in A. Brand, L. Allen, M. Altman, M. Hlava, J. Scott, *Learn. Publ.* **2015**, *28*, 151–155. This taxonomy provides a detailed classification of the various roles performed by each author.

The degree of contribution is coded as lead (black), equal (dark grey), or supporting (light grey).

	S. L.-A.	P. M.-M.	L. R.-M.	G. S.	J. R. C.	J.-D. M.	M. J. M.	J. L. M.	M. E. V.
**Conceptualization**. Ideas; formulation of overarching research goals and aims.						equal	equal	lead	lead
**Methodology**. Development or design of methodology; creation of models.	supporting	supporting	supporting	supporting	equal	equal	equal	equal	equal
**Validation**. Verification of the overall reproducibility of results and research outputs.	equal	equal	equal	equal	equal	equal	equal	equal	equal
**Formal Analysis**. Application of formal techniques to analyze the data.	equal	equal	equal	equal	equal	equal	equal		
**Investigation**: peptide synthesis and characterization; metal binding; in vitro KRAS inhibition studies.	lead				supporting				
**Investigation**: NMR spectroscopy.		lead					lead		
**Investigation**: Computational studies.			equal	equal					
**Resources**. Provision of reagents, materials, instrumentation, and analysis tools.						equal	equal	equal	equal
**Data Curation**. Management to annotate, scrub and maintain research data.	equal	equal	equal	equal	equal				
**Writing: Original Draft**. Preparation of the paper, specifically writing the initial draft.						equal	equal	equal	equal
**Writing: Review & Editing**. Critical review, commentary or revision.	equal	equal	equal	equal	equal	equal	equal	equal	equal
**Visualization**. Preparation, of the paper, specifically visualization &/or presentation.					equal	equal	equal	equal	lead
**Supervision**. Oversight responsibility for the research planning & execution.						equal	equal	equal	equal
**Project Administration**. Management and coordination of the research.									lead
**Funding Acquisition**. Acquisition of the financial support leading to this publication.						equal	equal	equal	equal

Supplementary Methods

Abbreviations: *Ahx*: 6-Aminohexanoic acid; *CARA*: Computer aided resonance assignment; *CNS*: Crystallography and NMR system; *DEDTC*: sodium diethyldithiocarbamate; *DIC*: *N,N′*-Diisopropylcarbodiimide; *DIEA*: *N,N*-diisopropylethylamine; *DMEM*: Dulbecco's Modified Eagle Medium; *HATU*: 2-(1*H*-7-aza-benzotriazol-1-*yl*)-1,1,3,3-tetramethyluronium hexafluorophosphate; *NMR*: Nuclear Magnetic Resonance; *NOESY*: Nuclear Overhauser Effect Spectroscopy; *SDS*: sodium dodecyl sulfate; *TFA*: trifluoroacetic acid; *TIS*: triisopropylsilane; *TMR*: 5-carboxytetramethylrhodamine; *TOCSY*: Total Correlation SpectroscopY; *p-ABA*: 4-acetamidobenzoic acid

Reagents: *cis*-$PdCl_2$(en) were purchased from *Aldrich*. TMR fluorophore were purchased from *Carbosynth* as a 5-Carboxytetramethylrhodamine succinimidyl ester. The antibodies ERK2 and phospho-ERK1/2 was kindly provided by Dr. José Costoya, from CiMUS–Centro Singular de Investigación en Medicina Molecular y Enfermedades Crónicas. Avenida Barcelona, s/n, 15782, Santiago de Compostela. Spain. All peptide synthesis reagents and amino acid derivatives were purchased from Sigma Aldrich and Iris Biotech; amino acids were purchased as protected Fmoc amino acids with the standard side chain protecting scheme: Fmoc-Ala-OH, Fmoc-Leu-OH, Fmoc-Lys(Boc)-OH, Fmoc-Ser(*t*-Bu)-OH, Fmoc-Glu(O*t*-Bu)-OH, Fmoc-Trp(Boc)-OH, Fmoc-Asn(Trt)-OH, Fmoc-Ile-OH, Fmoc-Thr(*t*-Bu)-OH, Fmoc-Arg(Pbf)-OH, Fmoc-His(Trt)-OH and Fmoc-Asp(O*t*-Bu)-OH. All other chemicals were purchased from *Aldrich* or *Fluka*. All solvents were dry and synthesis grade, unless specifically noted.

Peptide synthesis. The peptide **αH-His$_2$** was synthesized on a *Liberty Blue Lite* automatic microwave assisted peptide synthesizer from *CEM* Corporation, following the manufacturer's recommended procedures. The synthesis was on a 0.1 mmol scale using a 0.5 mmol/g load *H-Rink* amide *ChemMatrix* resin. Amino acids were coupled in 5-fold excess using DIC (*N,N′*-Diisopropylcarbodiimide) as activator, Oxime as base, and DMF as solvent. Couplings were conducted for 4 min at 90 °C. Deprotection of the temporal Fmoc protecting group was performed with 20% piperidine in DMF for 1 min at 75 °C. TMR was manually coupled to the N-terminus of the sequence by incubation of the resin-bound peptide with 3 eq. of 5-carboxytetramethylrhodamine (0.15 mmol, 64.5 mg), 3 eq. of HATU, and 5 eq. of DIEA 0.2 M in DMF for 60 min. Cleavage/deprotection was done by treating the resin-bound peptide for 2 h with 900 µL TFA, 50 µL CH_2Cl_2, 25 µL H_2O and 25 µL TIS (1 mL of cocktail/40 mg resin). The resin was filtered, and the filtrate was added onto ice-cold diethyl ether. After ~20 min, the precipitate was centrifuged and washed again with of ice-cold ether. The solid residue was dried under argon and redissolved in water/CH_3CN (1:1) for HPLC purification on a semipreparative *Agilent* 1100 series LC using a *Phenomenex Luna–C_{18}* (250 × 10 mm) reverse-phase column and a linear gradient (5 to 75% B over 40 min, 4 mL/min.; A: H_2O 0.1% TFA, B: CH_3CN 0.1% TFA). Collected fractions with pure products were freeze-dried to afford the desired peptide. The peptide was analyzed by analytical UHPLC-MS with an *Agilent 1200* series LC/MS using a *SB C_{18}* (1.8 µm, 2.1 × 50 mm) analytical column from *Phenomenex* with a linear gradient from 5 to 95% of solvent B for 20 min at a flow rate of 0.35 mL/min (A: water with 0.1% TFA, B: acetonitrile with 0.1% TFA). Compounds were detected by UV absorption at 222, 270, and 330 nm. Electrospray Ionization Mass Spectrometry (ESI/MS) was performed with an *Agilent 6120 Quadrupole* LC/MS model in positive scan mode using direct injection of the purified peptide solution into the MS detector.

Protein Expression and Purification. The constructs *pDNA2.0 6H-TEV-KRAS*, *pDNA2.0 6H-TEV-KRAS G12V* and *pDNA2.0 6H-FLAG-TEV-KRAS G12C* were purchased from *Addgene*. BL21 cells (*Invitrogen*) were transform with the constructs and grown in Luria broth (LB) to OD 600 0.7 and induced with 250 mM isopropyl β-D-1-thiogalactopyranoside (IPTG) for 16 h at 16 °C. Cells were pelleted and resuspended in lysis buffer (20 mM sodium phosphate, pH 8.0, 500 mM NaCl, 10 mM imidazole, 1 mM 2-mercaptoethanol (BME), 5% (vol/vol) glycerol). After centrifugation, the pellet was frozen until use. Then, protease inhibitor (EDTA free) and lysozyme (1mg/mL) were added, and the pellet was sonicated for 6 min. (35 sec. on /10 sec. off). Protein was purified over an IMAC (immobilized metal affinity chromatography) following *HisPur* Ni-NTA protocols (*Thermo Fisher*). Protein was concentrated in a 10-kDa Amicon Ultra-15 (*Millipore*) aliquoted with 15% glycerol, and then flash-frozen and stored at –20 ºC.

Nucleotide Release experiment. Loading of fluorescent-labeled GDP (mantGTP) to KRAS^{wt} was conducted following previous reports.[1,2] Purified KRAS^{wt} was buffer-exchanged in *Zeba* Spin Desalting Columns (*Thermo Fisher*) in loading buffer (20 mM Tris-HCl [pH 7.5], 50 mM NaCl, 4 mM EDTA and 1 mM

DTT). The resulted eluted KRAS^{wt}, after measuring their concentration, was incubated with 10-fold molar excess of mantGTP (*Abcam*) for 1.5 h at 20 °C in the dark. Reactions were supplemented with 10 mM $MgCl_2$ and incubated for 1 h on ice. Free nucleotide was removed using a *Zeba* Spin Desalting Columns (*Thermo Fisher*) that had been equilibrated with the reaction buffer (20 mM Tris-HCl, pH 7.5, 50 mM NaCl, 1 mM $MgCl_2$ and 1 mM DTT). The effect of peptides on the intrinsic rate of nucleotide release was monitored using the decrease in fluorescence with time as mantGTP dissociates from KRAS in a 100 µL reaction mixture (96-well plate) of 1 µM KRAS^{wt}/mantGPT complex and 10 µM of **αH-His$_2$** or **αH-His$_2$**[Pd]. Fluorescence was excited at 370 nm and emission was monitored at 430 nm during 30 min, using a *Tecan* Infinite M Plex plate reader. The data was processed using the program *GraphPad Prism*.

Cell internalization and inhibition of ERK1/2. All steps were performed on a sterile clean bench Telstar AV-100 at rt. Solutions stored in a fridge were warmed beforehand in a water bath (37 °C). Unless otherwise specified, all incubations were performed in DMEM containing 5% of fetal bovine serum (FBS-DMEM). Cell Culture: A549 cell line was cultured in DMEM (Dulbecco´s modified Eagle´s medium), 5 mM glutamine, penicillin (100 units/mL) and streptomycin (100 units/mL), all from *Invitrogen*. Proliferating cultures were maintained in a 5% CO_2 humidified incubator at 37 °C. For all the experiments, cells were seeded in the corresponding well at the indicated concentration two days before treatment. A549 cells were seeded on glass-bottom plates 48 h (150.000 cell/ml) before treatment. Culture medium was removed and DMEM containing 5% fetal bovine serum (FBS-DMEM) and **αH-His$_2$** or **αH-His$_2$**[Pd] (10 *µ*M) were added. The metallopeptide was made just before the addition to cells by pre-incubating with metal complexes (1:1) in water for 10 min. After 30 min of incubation with cells, these were washed twice with PBS and replaced with fresh FBS-DMEM to observe under the microscope with adequate filters. Digital pictures of the different samples were taken under identical conditions of gain and exposure. A549 cells were seeded on glass-bottom plates (150.000 cell/ml) 48 h before treatment. Culture medium was removed, the cells was serum-starved and **αH-His$_2$**, **αH-His$_2$**[Pd] or *cis*-$PdCl_2$(en) were added at different concentrations and incubated for 4 h. After two washes with PBS, the cells were lysed in Laemmli buffer containing Tris pH 6,8 1M, Glycerol, SDS, Bromophenol Blue and β-mercaptoethanol, and heated at 95 ºC for 10 min. The samples were separated by SDS-12.5% polyacrylamide gel and transferred to nitrocellulose membrane, to probed by Western blotting. Levels of total ERK2 and phosphorylated ERK were detected with anti-ERK1/2 and phospho-ERK1/2) antibodies, respectively and revealed with Luminata™ Classico Western HRP substrate following manufacter´s protocol The visualization was performed with ChemiDoc MP imaging system by *Biorad*, and the processing of the images and densitometry quantification was carried out with ImageLab program.

Fluorescence microscopy. All images were obtained with an *Andor* Zyla mounted on a *Nikon TiE.* LED excitation wavelength 550 nm. Filter cube TRITC-B-000 (Semrock): BP 543/22 nm, LP 593/40 nm and DM 562 nm. Images were further processed with *Image J*.

UHPLC-MS αH-His2

αH-His2 *TMR*-Ahx-RRFFGI**H**LTN**H**LKTEEGN, Ahx = aminohexanoic acid; TMN 5-carboxytetramethyl rhodamine.

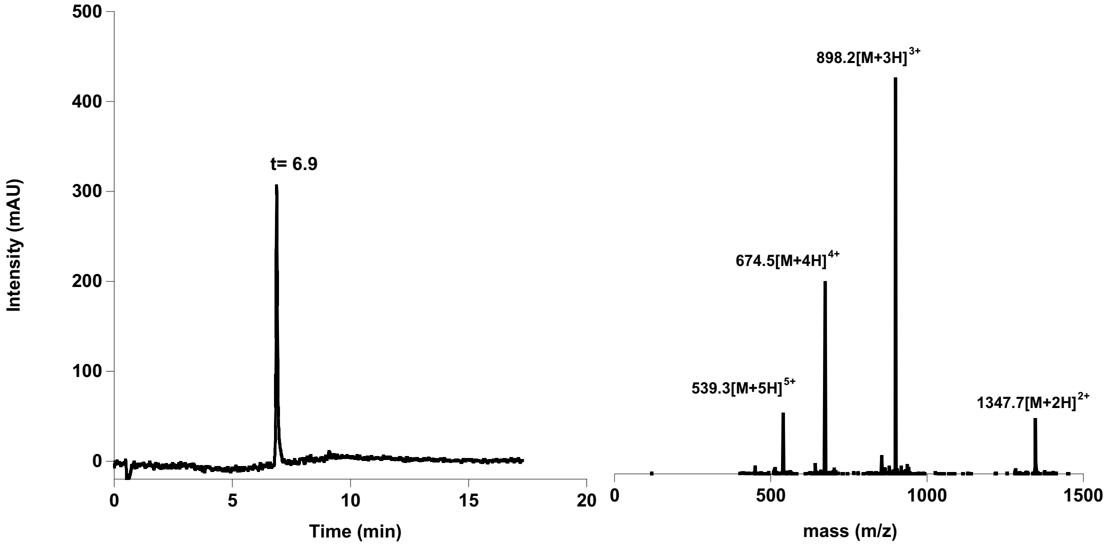

Fig. S1 *Left*: HPLC chromatogram of purified peptide. Gradient 5 to 95% B over 30 min. *Right*: Mass spectrum of the purified peptide. EM–ESI+ (m/z): Calcd. for $C_{127}H_{181}N_{35}O_{31}$: 2692.4. Found: 1347.7 $[M+2H]^{2+}$; 898.2 $[M+3H]^{3+}$; 674.5 $[M+4H]^{4+}$; 539.3 $[M+5H]^{5+}$

UHPLC-MS αH-His2[Pd]. The peptide was mixed with *cis*-$PdCl_2$(en) (1:1) in water for 10 min, and the resulting crude was analyzed by UHPLC-MS to corroborate the formation of metallopeptide.

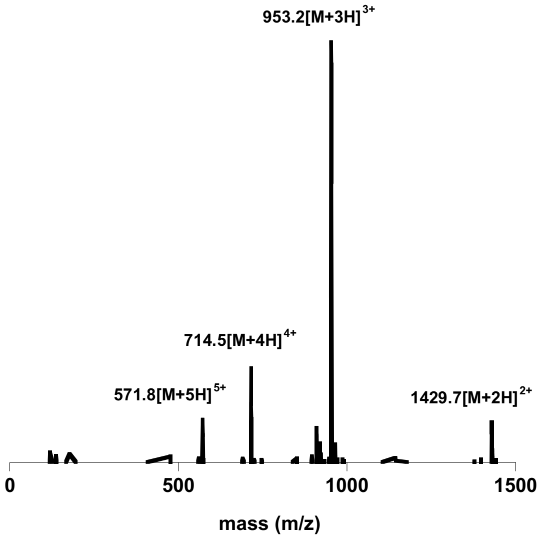

Fig. S2 Mass spectrum of the crude resulting from mixing **αH-His2** + *cis*-$PdCl_2$(en) (1:1), consistent with the formation of a complex containing Pd and the ethylendiamine ligand. EM–ESI+ (m/z): Calcd. for the metallopeptide $C_{129}H_{189}N_{37}O_{31}Pd$: 2858.34. Found: 1429.7 $[M+2H]^{2+}$; 953.2 $[M+3H]^{3+}$; 714.5 $[M+4H]^{4+}$; 571.8 $[M+5H]^{5+}$

Circular Dichroism. Circular Dichroism was measured on a *Jasco-715* coupled with a thermostat *Nestlab* RTE-111 using a 2 mm *Hellma* cuvette at 25 °C and the following settings: Acquisition range, 300-195 nm; band width, 2.0 nm; resolution, 0.2 nm; accumulation, 5 scans; sensitivity, 10 mdeg; response time, 0.25 s; speed, 100 nm/min. The spectra are the average of 5 scans and were processed using the "smooth" macro in the program *KaleidaGraph* (v 3.5 by Synergy Software).

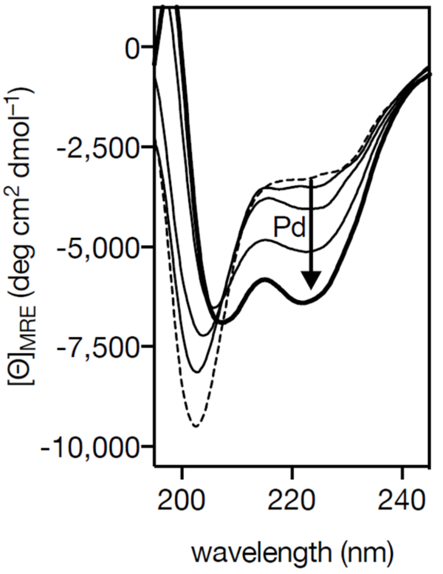

Fig S3. *Left:* CD titration experiment**.** Circular dichroism of a 20 μM solution of **αH-His$_2$** (dashed line) and addition of 1, 2, 5 and 10 equivalents of *cis*-$PdCl_2$(en). The experiments were carried out in 10 mM phosphate buffer pH 7.5, 100 mM of NaCl and 10% TFE, 25 °C. Mean residue molar ellipticity ($[\Theta]_{MRE}$) was calculated considering the peptide as an 18-mer.

Computational simulations. Ligand-protein docking exploration with GOLD, and GaudiMM, was first performed to discern the possible coordination modes of the Pd(II) complex with the **αH-His$_2$** peptide. Then, the **αH-His$_2$**[Pd] cores were parametrized with the Amber tool MCPB.py,[3] based on the bonded model approach. QM calculations were performed with the DFT formalism as implemented in Gaussian 09, combined with the hybrid functional B3LYP.[4] The basis set employed was 6-31g(d,p) for C, H and N atoms, while the SDD basis set with *f*-polarization function was used for the metal ion, with a pseudo-potential for the core electrons.[5] All these calculations were combined with the Grimme's D3 correction for dispersion.[6] The complexes were embedded in a solvent-polarizable dielectric continuum model (SMD, water).[7] Once these QM calculations were obtained, the parameters were created for those atoms coordinating the Pd(II) ion through Seminario's approach.[8] The point charges were computed based on the RESP protocol.[9] Once the parameters were obtained, the systems were set up with AMBER18 *tleap* to build the topology and the coordinates file. The force field used for the protein atoms was the ff14SB,[10] improved force field recommended for the simulation of peptides. For the remaining atoms, the GAFF force field was applied; while the parameters obtained from the MCPB.py calculation were used for the Pd(II) ion. Explicit water TIP3P solvent was also considered in the complexes,[11] introducing neutrality by the addition of three and one chloride ions for the metallic and free peptide, respectively. The systems were embedded into a cubic box containing between 2700 and 3000 water molecules, 10 Å from the protein to the edge of the box. The topology and the coordinates files achieved for each complex were then used for a first 10 ns classical Molecular Dynamics (MD) simulation. The coordinates of the last structure were used for the Gaussian accelerated Molecular Dynamics (GaMD) simulations,[12] which allows an extensive exploration of the conformational space. The AMBER ff14SB force field in an NVT ensemble was also used with SHAKE algorithm. A boost on both dihedral a total potential energy was applied, producing GaMDs of at least 2 µs for each system. To prove the correct exploration of the conformational space, PCA analysis were applied to the trajectories. Despite GaMD allows extensive conformational exploration, simulations were started from different putative folded geometries of the peptide to assure unbiased predictions to evaluate the progression of the system from the two extreme situations (data not shown).

a) b) c)

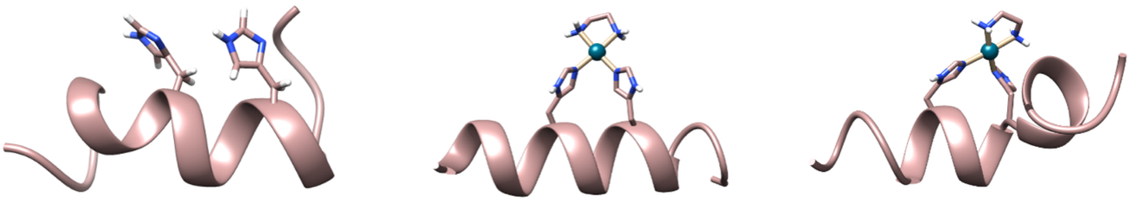

Fig S4. representative structures from each GaMD simulation.

A

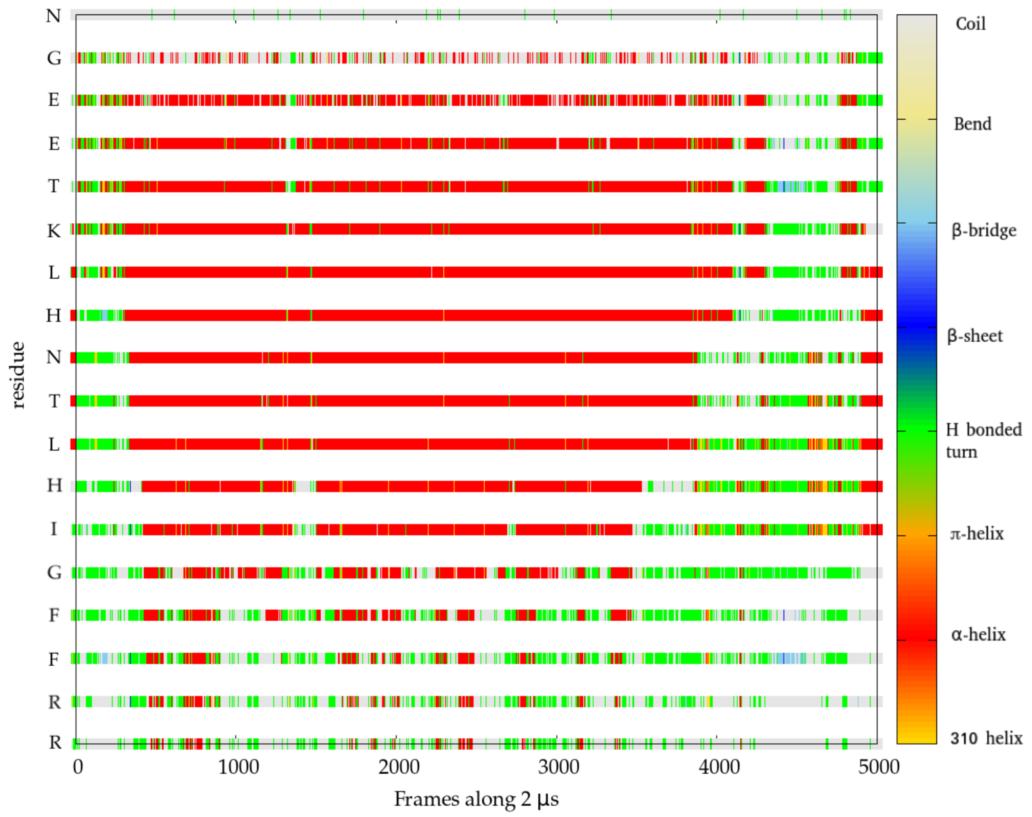
N
G
E
E
T
K
L
H
N
T
L
H
I
G
F
F
R
R
residue
0
1000
2000
3000
4000
5000
Frames along 2 µs
Coil
Bend
β-bridge
β-sheet
H bonded turn
π-helix
α-helix
310 helix

B

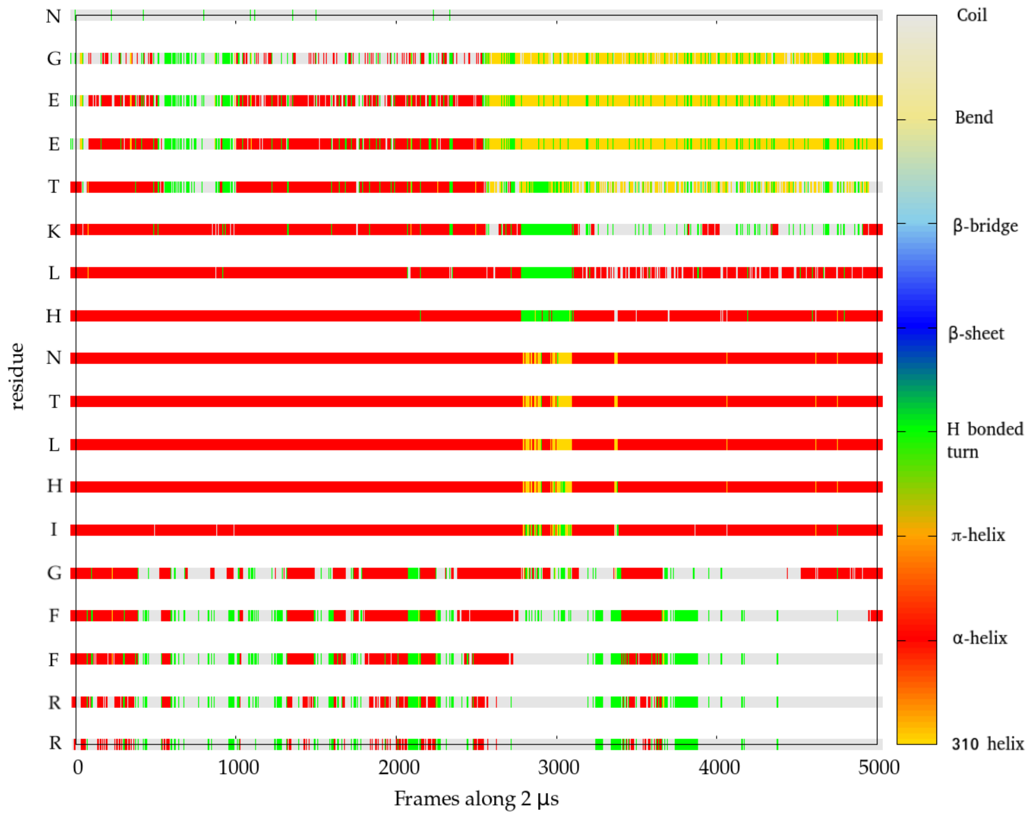
N
G
E
E
T
K
L
H
N
T
L
H
I
G
F
F
R
R
residue
0
1000
2000
3000
4000
5000
Frames along 2 µs
Coil
Bend
β-bridge
β-sheet
H bonded turn
π-helix
α-helix
310 helix

c

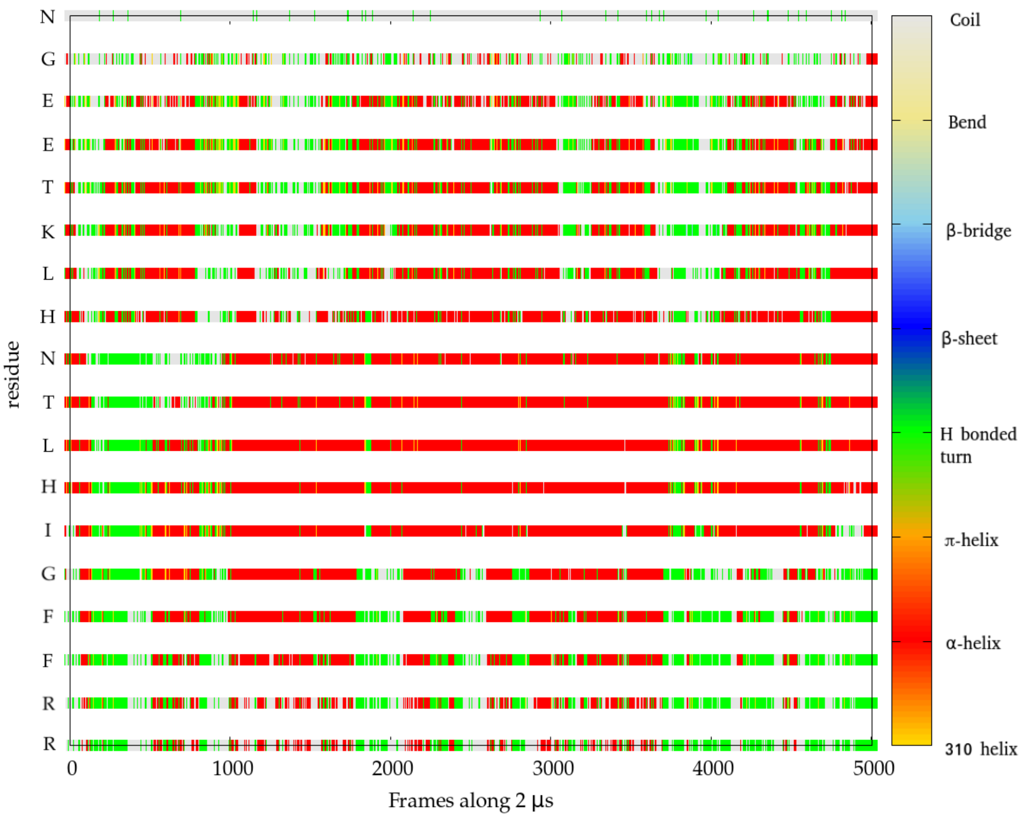

Fig S5. Secondary Structure Analysis along 2μs GaMD trajectory: a) **αH-His₂**; b) **αH-His**[Pd] ***N*τ-His933/*N*τ-His937** complex; c) **αH-His**[Pd] ***N*τ-His933/*N*π-His937**

Fluorescence Anisotropy and curve fitting. Anisotropy measurements were made with a *Jobin-Yvon Fluoromax-3*, (DataMax 2.20), coupled to a *Wavelength Electronics* LFI–3751 temperature controller, and using a 1 mL *Hellma* micro cuvette. Settings: integration time, 2.0 s; excitation slit width, 5.0 nm; emission slit width, 20.0 nm; excitation wavelength, 559 nm; emission wavelength, 585 nm. Aliquots of a stock solution of protein were added onto 15 nM solutions of **αH-His$_2$** or **αH-His$_2$**[Pd] in Tris-HCl buffer 20 mM, pH 7.5, 100 mM NaCl, and the anisotropy was recorded after each addition. Experimental data correspond to the mean of three independent titration experiments and are representative of at least three biological replicates performed with independent preparations of recombinant proteins. All experiments carried out at 25 °C. Before use the corresponding purified protein, the glycerol was removed using a *Zeba* Spin Desalting Columns (*Thermo fisher*) that had been equilibrated with anisotropy buffer (Tris-HCl buffer 20 mM, pH 7.5, 100 mM NaCl). For the switch experiment, after completing the titration at saturating concentrations of the protein, 50 eq. of DEDTC (relative to the metallopeptide) was added to the solution and the anisotropy was recorded. After that, 50 eq. of *cis*-$PdCl_2$(en) was added (relative to the metallopeptide) and the anisotropy was recorded again.

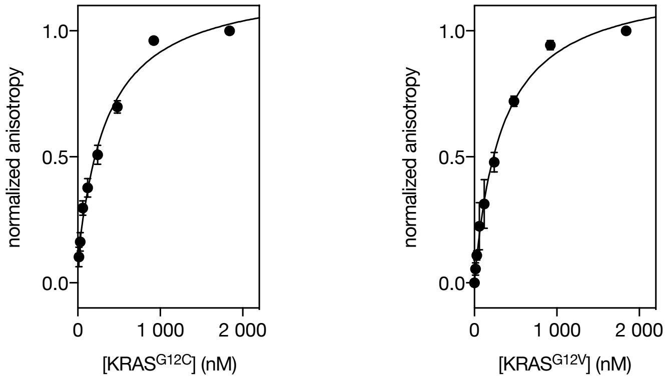

Fig S6. Anisotropy titration with oncogenic mutants KRAS^{G12C} and KRAS^{G12V}. Normalized emission anisotropy at 559 nm of a 15 nM solution of **αH-His$_2$**[Pd] with increasing concentrations of KRAS^{G12C} (*Left*) and KRAS^{G12V} (*Right*). The best fit to a 1:1 binding model is also shown. Data are mean ± SEM for experiments performed in technical triplicate and are representative of at least three biological replicates performed with independent preparations of recombinants KRAS. All experiments carried out at 25 °C.

Experimental data were fitted with the *DynaFit 4.0* software, which performs a numerical treatment of the system.[13,14] The program is available free of charge for academia at http://www.biokin.com/dynafit/. *Dynafit* requires plain text files (scripts) that contains information about the chemical model underlying the experimental data, the values of model parameters, such as starting concentrations of reactants, as well as information about location of the files. A typical script used in the analysis titrations is included below. The file has been commented to indicate the purpose of the keywords and sections, but the reader is recommended to review the *DynaFit* scripting manual distributed along the program or available at the *DynaFit* website.

```
[task]                        ;semicolons indicate comments from actual instructions
 task = fit                   ;nature of the calculation to be performed
 data = equilibria

[mechanism]                   ;Free-form 1:1 binding model with Kd
 R + L <==> RL : Kd dissoc    ;to be calculated as dissociation constant

[constants]                   ;Initial Kd value for iteration
 Kd = 1.0 ?                   ;the "?" indicates that this will be optimized

[concentrations]
 R = 2.0                      ;Fixed conc. of the DNA during the peptide titration

[responses]                   ;contribution to the spectroscopic signal of each
 R = 0.1 ?                    ;of the different components of the equilibrium
 RL = 1.5 ?                   ;these will be optimized ("?" after the values).

[data]                        ;location of files and information about the data
 variable  L                  ;the species that changes conc. during the titration
 offset auto ?
 directory ./exp/brHis        ;file path (relative to DynaFit program location)
 extension txt
 file f1                      ;name of the experimental data file

[output]
 directory ./exp/brHis/out    ;path indicating location of DynaFit output files

[settings]                    ;cosmetic settings that control DynaFit graphics
 {Output}                     ;fits were exported & finally plotted with
 XAxisUnit = uM               ;GraphPad Prism 7.0c. GraphPad Software,
 BlackBackground = n          ;La Jolla California, www.graphpad.com
 XAxisLabel = [peptide]
 YAxisLabel = anisotropy
 WriteTXT = y
```

High resolution NMR experiments. NMR experiments were recorded on a *Bruker* Avance III 600-MHz spectrometer (IRB Barcelona) equipped with a quadruple (^{1}H, ^{13}C, ^{15}N, ^{31}P) resonance cryogenic probe head and a z-pulse field gradient unit at 298 K using a 1 mM solution of **αH-His$_2$** peptide—in the presence or absence of *cis*-$PdCl_2$(en)—1D proton spectra were recorded with a sweep width of 12000 Hz and 32 k data points. A total of 16 scans were accumulated with an acquisition time of 2.05 s. A Watergate w5 composite pulse was used to suppress the water signal. ^{1}H 2D-TOCSY and NOESY experiments were acquired in 90% H_2O/10% D_2O and used to assign the spin systems corresponding to the peptide resonances.[15–17] For the 2D-NOESY experiments, mixing times of 300, 150 and 80 ms were acquired to minimize the impact of spin-diffusion in the assignments. Spin-locking fields of 8 kHz and 50 ms mixing time was used for the 2D-TOCSY experiments. All 2D spectral widths were 8000 Hz. The data size was 512 points in F1, indirect dimension, and 2048 points in F2, direct dimension. For each F1 value, 48 transients were accumulated in the NOESY and 32 in the TOCSY experiments respectively. Data were processed with a combination of exponential and shifted sine-bell window functions for each dimension followed by automated baseline and phase correction using *TopSpin* 3.5 (©Bruker 2020). The 512 × 2 k data matrices were zero-filled to 2k×2k (NOESY and TOCSY). Structure Calculation: To prove that the primary structure (composition and connectivity) corresponds to the theoretical sequence, we have followed the sequence assignment strategy.[17] We identified the characteristic spin system of every residue in the sequence, using the 2D TOCSY experiment and every spin system was connected to the following one via NOEs observed from the side-chain of a given residue (*i*) to the amide proton of the following residue (*i*+1) as well as from the amide proton of (*i*) to the amide of (*i*+1). The full spin analysis as well as the assignment of the NOEs were carried out manually using CARA software.[18,19] Distance restraints derived from the NOESY experiments were used for the NMR-based model building of the peptide in solution using unambiguously assigned peaks exclusively and the program CNS 1.2 (Crystallography and NMR system).[20] The protocol consisted of an implicit water simulated-annealing of 120 structures using 8,000 cooling steps followed by an explicit water refinement of the calculated structures using all experimental restraints during 1200 steps. The Pd coordination was not explicitly included in the calculation. To display the metal coordination, we manually optimized the His rotamers to facilitate the coordination and the Pd was added to the final model displayed in Figure 2.

Chemical shift assignments

AlphaHHis2

1 ARG	**HN**	8.507
	**HA**	4.300
	**HB2**	1.763
	**HG2**	1.616
	**HG3**	1.529
	**HD2/3**	3.103
	**HE**	7.084
2 ARG	**HN**	8.249
	**HA**	4.180
	**HB2/3**	1.522
	**HG2/3**	1.303
	**HD2/3**	2.981
	**HE**	6.969
3 PHE	**HN**	8.089
	**HA**	4.516
	**HB2**	2.957
	**HB3**	2.807
	**HZ/QD**	7.053
	**QE**	7.160
4 PHE	**HN**	8.131
	**HA**	4.519
	**HB2**	3.048
	**HB3**	2.860
	**HZ**	7.182
	**QD**	7.137
	**QE**	7.234
5 GLY	**HN**	7.841
	**HA1**	3.762
	**HA2**	3.701
6 ILE	**HN**	7.815
	**HA**	4.044
	**HB**	1.710
	**QG2**	1.032
	**HG12**	1.236
	**QD1**	0.739
7 HIS	**HN**	8.564
	**HA**	4.685
	**HB2**	3.148
	**HB3**	3.043
	**HD2**	7.149
	**HE1**	8.445
8 LEU	**HN**	8.297
	**HA**	4.320
	**HB2**	1.535
	**HB3**	1.470
	**QD1/2**	0.745
9 THR	**HN**	8.134
	**HA**	4.228
	**HB**	4.086
	**QG2**	1.051
10 ASN	**HN**	8.311
	**HA**	4.582
	**HB2**	2.679
	**HB3**	2.669
	**HD21**	7.502
	**HD22**	7.062
11 HIS	**HN**	8.440
	**HA**	4.590
	**HB2**	3.184
	**HB3**	3.063
	**HD2**	7.182
	**HE1**	8.510
12 LEU	**HN**	8.180
	**HA**	4.252
	**HB2**	1.549
	**HB3**	1.470
	**QD1/2**	0.787
13 LYS	**HN**	8.188
	**HA**	4.271
	**HB2**	1.776
	**HB3**	1.696
	**HG2/3**	1.343
14 THR	**HN**	8.126
	**HA**	4.221
	**HB**	4.138
	**QG2**	1.106
15 GLU	**HN**	8.312
	**HB2**	2.015
	**HB3**	1.891
	**HG2/3**	2.266
16 GLU	**HN**	8.299
	**HB2/3**	1.929
	**HG2/3**	2.274
17 GLY	**HN**	8.349
	**HA1/2**	3.861
18 ASN	**HN**	8.237
	**HA**	4.634
	**HB2**	2.737
	**HB3**	2.654
	**HD21**	7.477
	**HD22**	6.819

Chemical shift assignments for AlphaHHis2 + 3 Equiv *cis*-$PdCl_2$(en)

1 ARG	**HN**	8.504
	**HA**	4.293
	**HB2/3**	1.768
	**HG2**	1.606
	**HG3**	1.524
	**HD2/3**	3.115
	**HE**	7.098
2 ARG	**HN**	8.231
	**HA**	4.175
	**HB2**	1.275
	**HB3**	1.273
	**HG2/3**	1.519
	**HD2/3**	2.978
	**HE**	6.982
3 PHE	**HN**	8.041
	**HA**	4.512
	**HB2**	2.960
	**HB3**	2.791
	**HZ/ QD**	7.035
	**QE**	7.160
4 PHE	**HN**	8.130
	**HA**	4.527
	**HB2**	3.056
	**HB3**	2.886
	**HZ/QD**	7.132
	**QE**	7.232
5 GLY	**HN**	7.892
	**HA1**	3.762
	**HA2**	3.700
6 ILE	**HN**	7.811
	**HA**	3.944
	**HB**	1.650
	**QG2**	0.667
	**HG12**	1.058
	**HG13**	0.958
	**QD1**	0.636
7 HIS	**HN**	8.447
	**HA**	4.371
	**HB2**	3.031
	**HB3**	2.956
	**HD2**	6.649
	**HE1**	7.537
8 LEU	**HN**	7.988
	**HA**	4.228
	**HB2**	1.684
	**HB3**	1.596
	**QD1**	0.814
	**QD2**	0.776
9 THR	**HN**	7.636
	**HA**	4.337
	**HB**	4.148
	**QG2**	1.057
10 ASN	**HN**	8.385
	**HA**	4.421
	**HB2**	2.761
	**HB3**	2.619
	**HD21**	7.048
	**HD22**	7.492
11 HIS	**HN**	8.045
	**HA**	4.897
	**HB2**	2.775
	**HB3**	2.727
	**HD2**	6.651
	**HE1**	7.611
12 LEU	**HN**	7.953
	**HA**	4.031
	**HB2**	1.556
	**HB3**	1.475
	**HG**	1.761
	**QD1/2**	0.811
13 LYS	**HN**	8.448
	**HA**	4.334
	**HB2**	1.758
	**HB3**	1.517
	**HG2/3**	1.386
	**QD**	1.602
	**HE2**	2.904
	**HZ1**	7.425
14 THR	**HN**	7.930
	**HA**	4.238
	**HB**	4.145
	**QG2**	1.100
15 GLU	**HN**	8.233
	**HA**	4.275
	**HB2**	2.030
	**HB3**	1.898
	**HG2/3**	2.346
16 GLU	**HN**	8.268
	**HB2**	2.033
	**HB3**	1.876
	**HG2/3**	2.355
17 GLY	**HN**	8.296
	**HA1**	3.866
	**HA2**	3.844
18 ASN	**HN**	8.217
	**HA**	4.447
	**HB2**	2.717
	**HB3**	2.653
	**HD21**	6.819
	**HD22**	7.470

NMR Spectra

KRAS peptide free

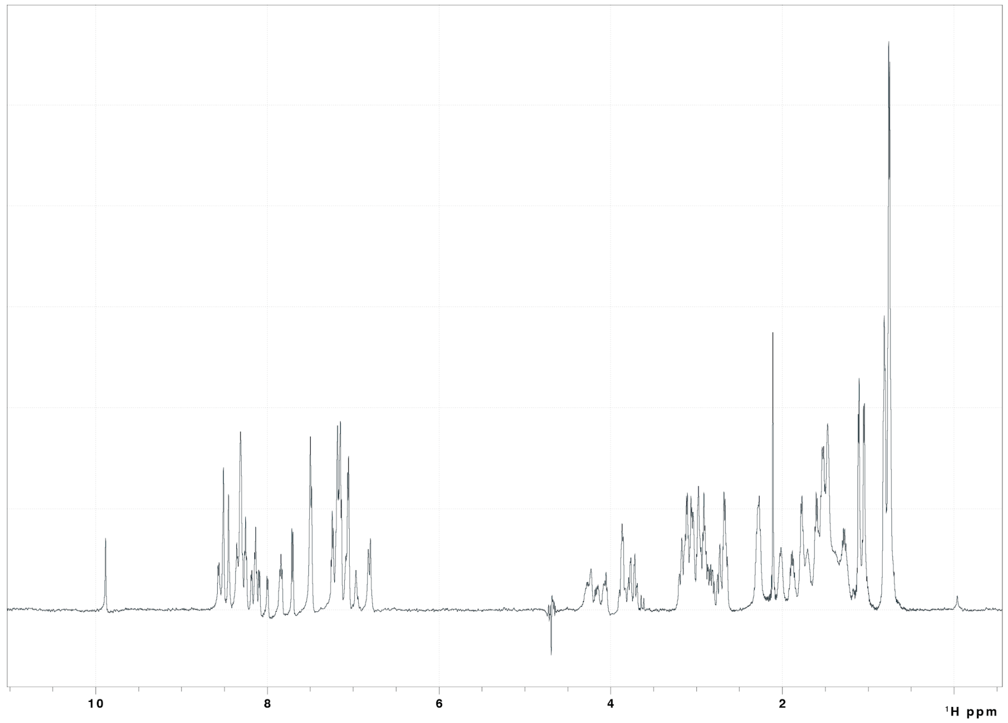

KRAS peptide free - TOCSY

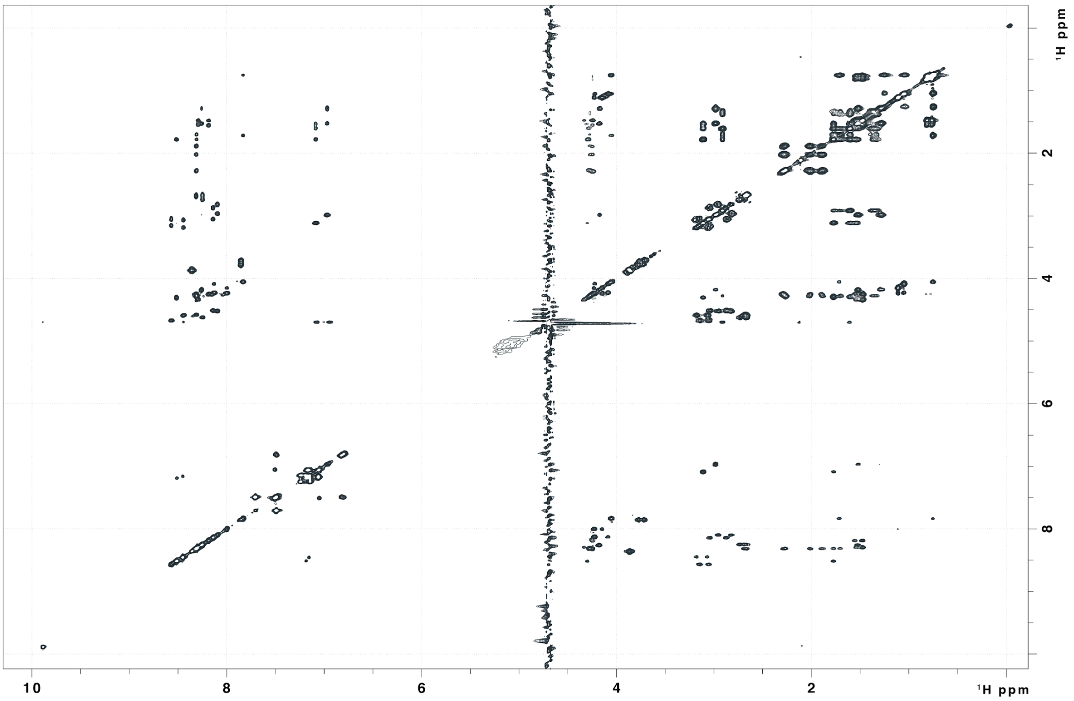

KRAS peptide free - NOESY

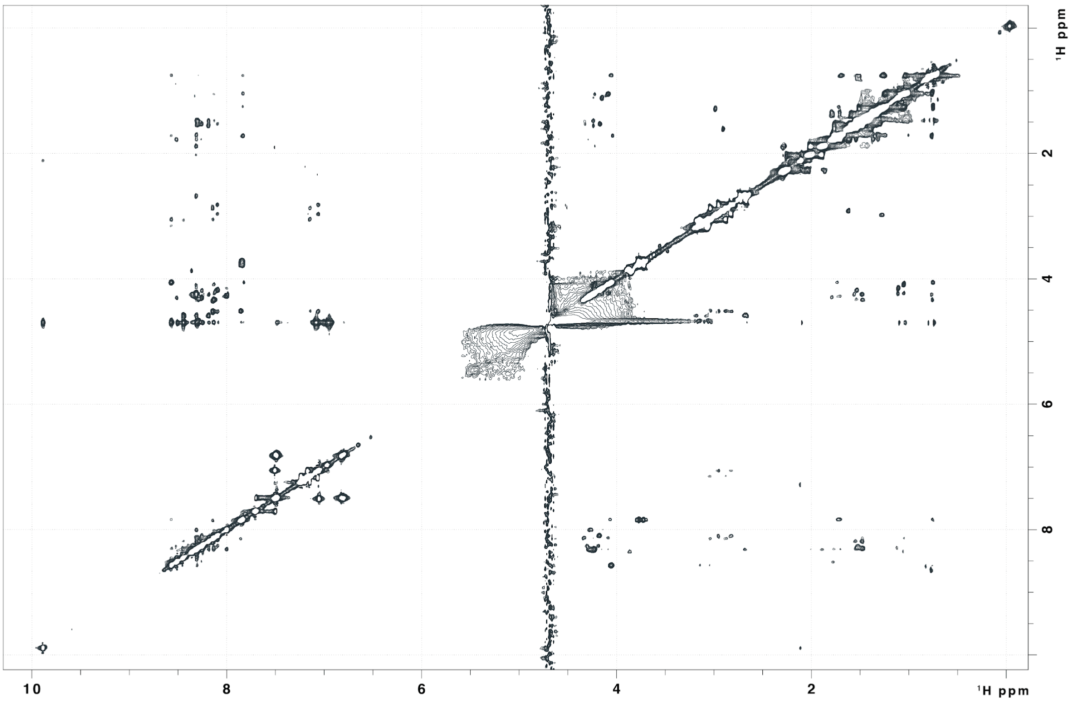

KRAS peptide free + 3eqv Pd

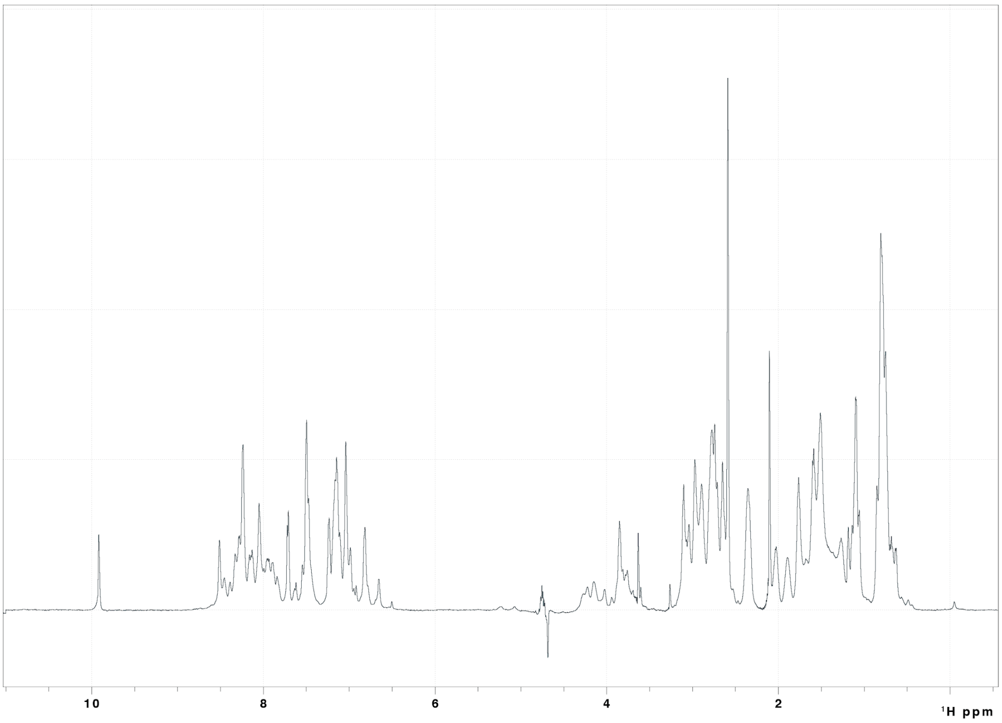

KRAS peptide free + 3eqv Pd - TOCSY

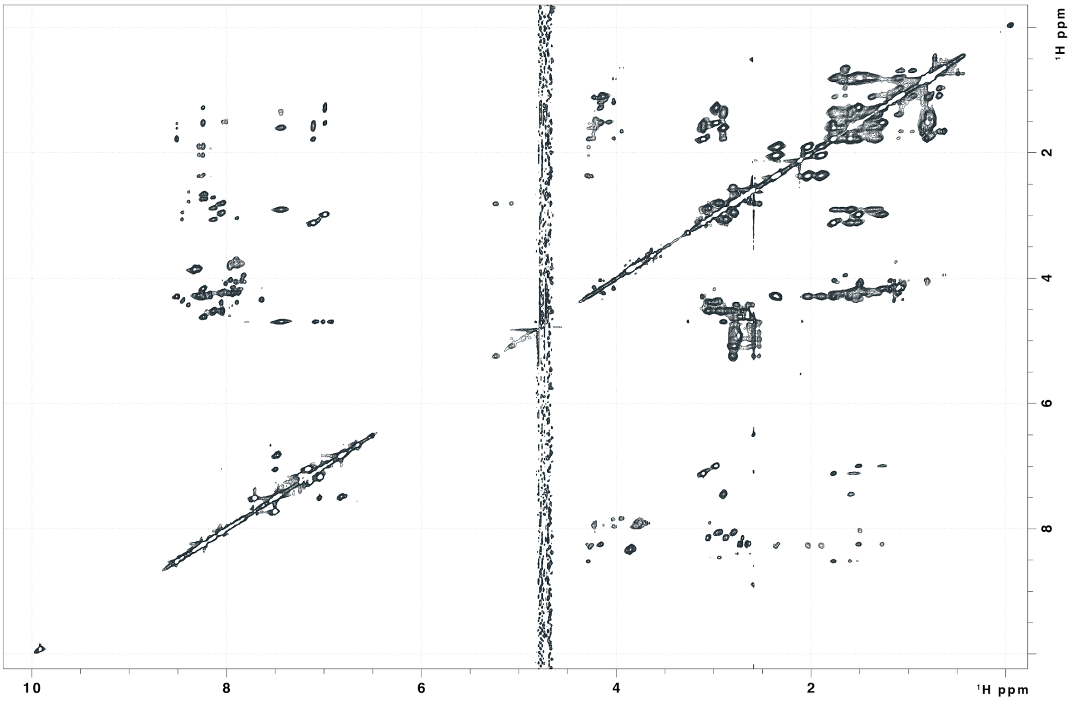

KRAS peptide free + 3eqv Pd - NOESY

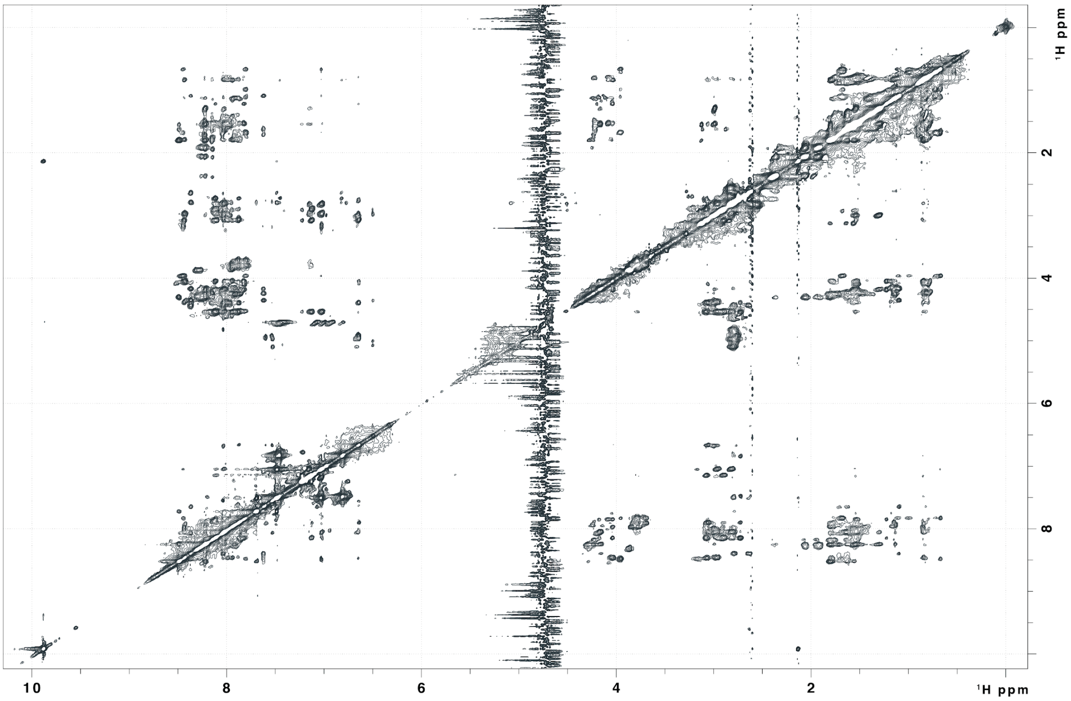

Stability studies. αH-His$_2$[Pd] was incubated for 4 h at 37 °C in DMEM + 5%FBS, in Hela cells lysates and in PBS. The solutions were then analyzed by HPLC-MS, showing that **αH-His$_2$**[Pd] remains coordinated and stable.

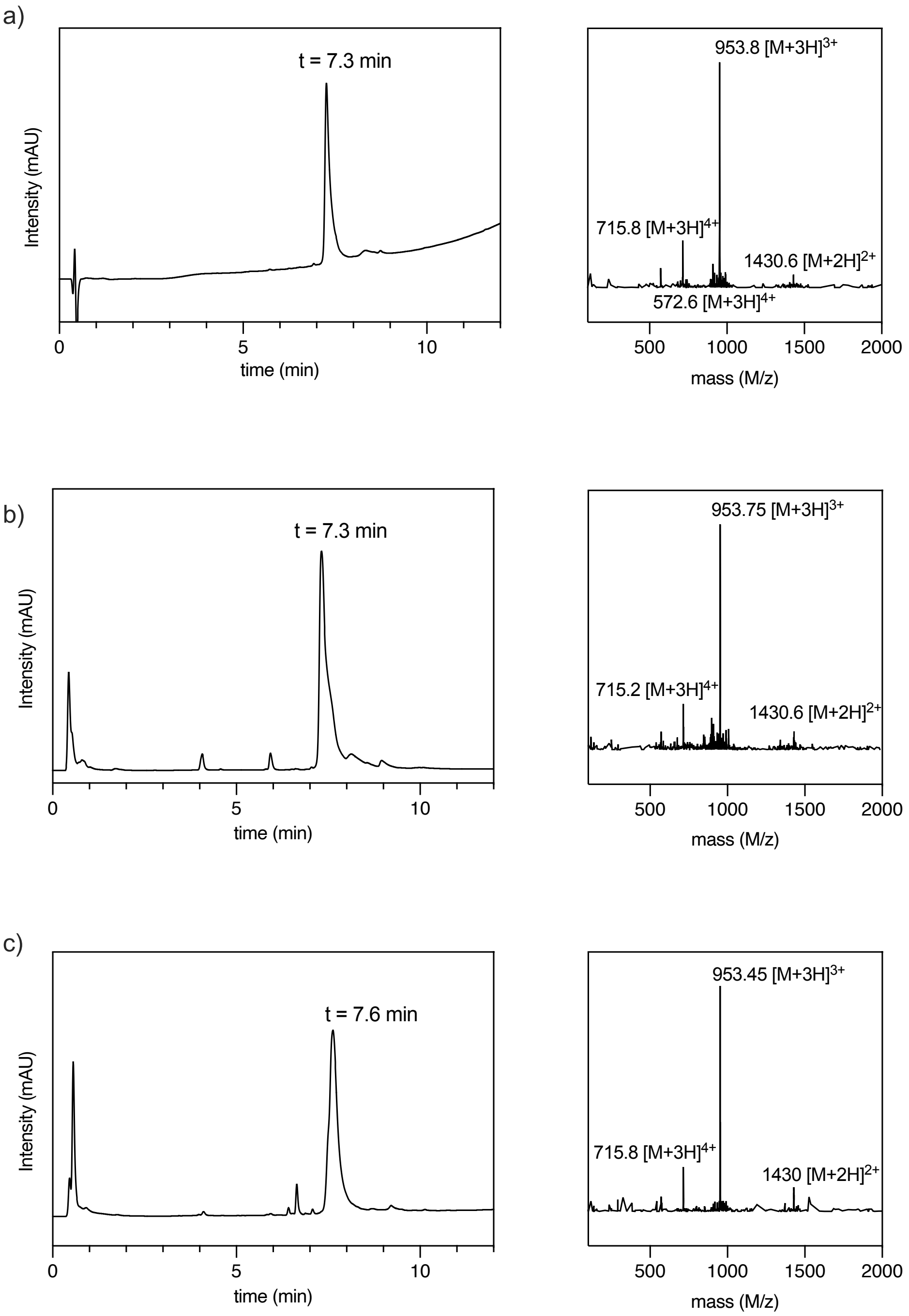

Fig S7. Stability study of **αH-His$_2$**[Pd]. a) incubated with PBS; b) incubated with DMEM + 5% FBS; c) incubated with HeLa cells lysates.

ICP-MS assays. For the ICP measurements, 100.000 cells per well were seeded in 24-well plate two days before treatment with metallopeptide in different concentrations (10 μM and 50 μM) for 4h. Cells were washed twice with PBS and lysed in 70% HNO_3. The lysates were digested in duplicate with HNO_3/H_2O_2 by microwave heating before being analyzed. After performed the ICP-MS we detected only a small percentage of palladium inside the cells.

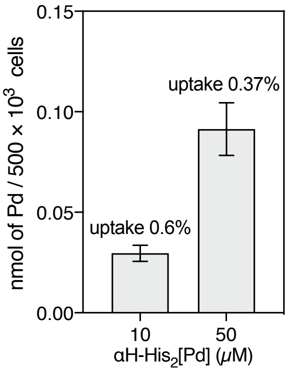

Figure S8. ICP-MS results on the intracellular accumulation of Pd**.** We incubated HeLa cells with 10 µM or 50 µM of metallopeptide for 4 h at 37 °C. Finally, the cells were washed two time with PBS and digested with HNO_3. Data are mean +/- SEM for experimental repeated in two independent times.

MTT Assays. The toxicity of metallopeptide, peptide and *cis*-Pd(en)Cl_2 was tested by MTT assays in HeLa cells as follows: 100,000 cells per well were seeded in 96 well plates two days before treatment with different concentrations of the peptides/metal. After 24 h of incubation, HEPES containing 3-(4,5-dimethylthiazol-2-yl)-2,5-diphenyl tetrazolium bromide (MTT) was added to the cell culture medium to a final concentration of 0.5 mg/ml. Cells were then incubated for 4 h to allow the formation of formazan precipitates by metabolically active cells. A detergent solution of 10% SDS (sodium dodecyl sulphate) and 0.01 M HCl was then added, and the plate was incubated overnight at room temperature to allow the solubilization of the precipitates. The quantity of formazan in each well (directly proportional to the number of viable cells) was measured by recording changes in absorbance at 570 nm in a microtiter plate reading spectrophotometer (*Tecan Infinite 200 PRO*). The MTT assays showed a moderate decrease on cell growth at high concentrations of metallopeptide.

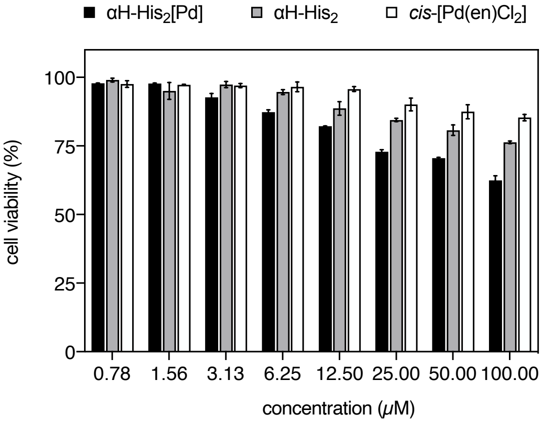

Figure S9. Toxicity assay. HeLa cells were incubated with peptide, metallopeptide and palladium at different concentrations, for 24 h at 37 °C. Then, the cells were washed two time with PBS before carrying out the MTT assay. Data are mean +/- SEM for experimental repeated in two independent times.

Supplementary References

1. Margarit, S. M. *et al.* Structural evidence for feedback activation by Ras.GTP of the Ras-specific nucleotide exchange factor SOS. *Cell* **112**, 685–695 (2003).

2. McCarthy, M. J. *et al.* Discovery of High-Affinity Noncovalent Allosteric KRAS Inhibitors That Disrupt Effector Binding. *ACS Omega* **4**, 2921–2930 (2019).

3. Li, P. & Merz, K. M., Jr. MCPB.py: A Python Based Metal Center Parameter Builder. *J. Chem. Inf. Model.* **56**, 599–604 (2016).

4. Becke, A. D. Density-functional thermochemistry. III. The role of exact exchange. *J. Chem. Phys.* **98**, 5648–5652 (1993).

5. Bergner, A., Dolg, M., Küchle, W., Stoll, H. & Preuß, H. Ab initio energy-adjusted pseudopotentials for elements of groups 13–17. *Mol. Phys.* **80**, 1431–1441 (1993).

6. Grimme, S., Antony, J., Ehrlich, S. & Krieg, H. A consistent and accurate ab initio parametrization of density functional dispersion correction (DFT-D) for the 94 elements H-Pu. *J. Chem. Phys.* **132**, 154104 (2010).

7. Marenich, A. V., Cramer, C. J. & Truhlar, D. G. Universal solvation model based on solute electron density and on a continuum model of the solvent defined by the bulk dielectric constant and atomic surface tensions. *J. Phys. Chem. B* **113**, 6378–6396 (2009).

8. Seminario, J. M. Calculation of intramolecular force fields from second-derivative tensors. *Int. J. Quantum Chem.* **60**, 1271–1277 (1996).

9. Bayly, C. I., Cieplak, P., Cornell, W. & Kollman, P. A. A well-behaved electrostatic potential based method using charge restraints for deriving atomic charges: the RESP model. *J. Phys. Chem.* **97**, 10269–10280 (1993).

10. Maier, J. A. *et al.* ff14SB: Improving the Accuracy of Protein Side Chain and Backbone Parameters from ff99SB. *J. Chem. Theory Comput.* **11**, 3696–3713 (2015).

11. Jorgensen, W. L., Chandrasekhar, J., Madura, J. D., Impey, R. W. & Klein, M. L. Comparison of simple potential functions for simulating liquid water. *J. Chem. Phys.* **79**, 926–935 (1983).

12. Miao, Y. *et al.* Improved Reweighting of Accelerated Molecular Dynamics Simulations for Free Energy Calculation. *J. Chem. Theory Comput.* **10**, 2677–2689 (2014).

13. Kuzmič, P. DynaFit—A Software Package for Enzymology. in *Methods in Enzymology* vol. 467 247–280 (Academic Press, 2009).

14. Kuzmic, P. Program DYNAFIT for the analysis of enzyme kinetic data: application to HIV proteinase. *Anal. Biochem.* **237**, 260–273 (1996).

15. Bax, A. & Davis, D. G. Practical aspects of two-dimensional transverse NOE spectroscopy. *J. Magn. Reson.* **63**, 207–213 (1985).

16. Macura, S. & Ernst, R. R. Elucidation of cross relaxation in liquids by two-dimensional N.M.R. spectroscopy. *Mol. Phys.* **41**, 95–117 (1980).

17. Wuthrich, K. *NMR of proteins and nucleic acids.* (John Wiley & Sons, 1986).

18. Ramirez-Espain, X., Ruiz, L., Martin-Malpartida, P., Oschkinat, H. & Macias, M. J. Structural characterization of a new binding motif and a novel binding mode in group 2 WW domains. *J. Mol. Biol.* **373**, 1255–1268 (2007).

19. Martín-Gago, P. *et al.* A tetradecapeptide somatostatin dicarba-analog: Synthesis, structural impact and biological activity. *Bioorg. Med. Chem. Lett.* **24**, 103–107 (2014).

20. Brunger, A. T. Version 1.2 of the Crystallography and NMR system. *Nat. Protoc.* **2**, 2728–2733 (2007).
